# Disulfidptosis: a new form of programmed cell death

**DOI:** 10.1186/s13046-023-02712-2

**Published:** 2023-05-31

**Authors:** Tingjin Zheng, Qingbo Liu, Feiyue Xing, Chong Zeng, Weidong Wang

**Affiliations:** 1 Department of Clinical Laboratory, Quanzhou First Hospital Affiliated to Fujian Medical University, Quanzhou City, 362000 Fujian China; 2 Department of Hepatobiliary Surgery, Shunde Hospital of Southern Medical University (The First People’s Hospital of Shunde), Foshan, 528300 Guangdong China; 3 Institute of Tissue Transplantation and Immunology, Department of Immunobiology, Jinan University, Guangzhou, Guangdong China; 4 Medical Research Center, Shunde Hospital, Southern Medical University, Foshan, 528300 Guangdong China

## Abstract

Disulfidptosis, a new form of cell death triggered by disulfide stress, is characterized by the collapse of cytoskeleton proteins and F-actin due to the intracellular accumulation of disulfides. This discovery will eventually aid in the development of therapeutic strategies against cancer.

**Supplementary Information:**

The online version contains supplementary material available at 10.1186/s13046-023-02712-2.

## Background

Disulfides are relatively stable products that maintain the secondary, tertiary, and quaternary structures of proteins by acting as inter- and intra-subunit cross-links; they confer physical and chemical stability to proteins [1, 2]. They are generated in response to oxidative stress; therefore, a better understanding of how disulfide accumulation causes cell death is of particular interest. A latest study published in the journal *Nature Cell Biology*, Liu et al. [3] shed light on a novel form of programmed cell death, “disulfidptosis” that is different from previously reported cell death forms, such as apoptosis, necroptosis, pyroptosis, autophagy, ferroptosis, and cuproptosis [4]. Excessive intracellular accumulation of disulfides in solute carrier family 7 member 11 (SLC7A11) high expression (SLC7A11^high^) cells under glucose starvation conditions and without repair mechanisms resulted in disulfide stress, which led to disulfidptosis, an unusual form of cell death with a specific underlying mechanism [3].

**Fig. 1:**
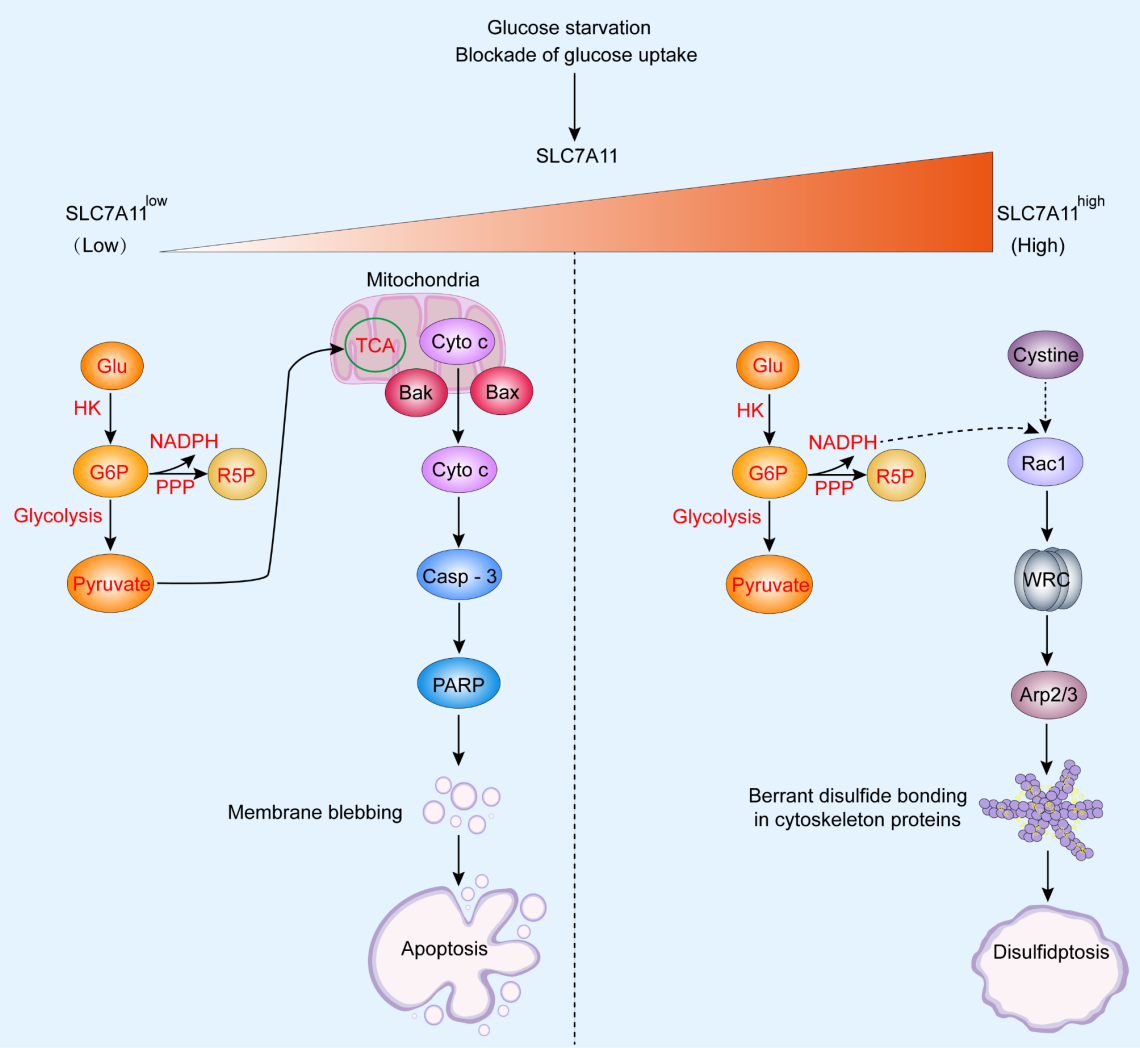
Schematic overview of the main difference between apoptosis and disulfidptosis under glucose starvation or blockade of glucose uptake in SLC7A11^low^ and SLC7A11^high^ cells. In SLC7A11^low^ cells, intracellular glucose level, formation of G6P, and formation of NADPH and pyruvate via the PPP and glycolysis, respectively, decreased under conditions of glucose starvation or blockade of glucose uptake. The formation of pyruvate through the TCA cycle and mitochondrial oxidative phosphorylation were inhibited. These changes up-regulated the expression of Bax and Bak, led to the release of cytochrome c from mitochondria, activated Casp-3, and promoted PARP-induced membrane blebbing and cell apoptosis. In SLC7A11^high^ cells, glucose starvation or blockade of glucose uptake led to high uptake of cystine and reduction to cysteine, NADPH depletion, disulfide stress caused by the intracellular accumulation of disulfides, which activated the Rac–WRC-Arp2/3 signaling pathway, contributing to aberrant disulfide bonds in actin cytoskeleton proteins and disulfidptosis. The red fonts indicate inhibition or reduction, and the black fonts indicates promotion or increase. Abbreviations: GLU, glucose; HK, hexokinase; PPP, pentose phosphate pathway; G6P, glucose-6-phosphate; R5P, ribulose-5-phosphate; Cyto c, cytochrome c; TCA, tricarboxylic acid; Bax, Bcl-2-associated X protein; BAK, BCL2 antagonist; NADPH, nicotinamide adenine dinucleotide phosphate; Casp-3, caspase-3; PARP, poly (ADP-ribose) polymerase; WRC, WAVE regulatory complex; Apr, actin-related protein

The activation of disulfidptosis might require three hallmarks: (1) high expression of SLC7A11, which imports extracellular cysteine and exports intracellular glutamate, resulting in high uptake of extracellular cysteine and excessive intracellular accumulation of cysteine contribute to disulfide stress in cell metabolism [5, 6]; (2) glucose starvation conditions that block glucose metabolism to generate the reduced form of nicotinamide adenine dinucleotide phosphate (NADPH) via the pentose phosphate pathway (PPP) [7]; and (3) formation of aberrant disulfide bonds between actin cytoskeleton proteins. When all these conditions are met, excessive accumulation of disulfides occurs, which contributes to disulfide bonding among actin cytoskeleton proteins, actin contraction, and detachment from the plasma membrane, ultimately leading to cell shrinkage and death [3]. However, a low expression of SLC7A11 combined with glucose starvation or blockade of glucose uptake decreases the intracellular level of glucose, inhibits the formation of glucose 6-phosphate (via hexokinase), and suppresses the formation of NADPH and pyruvate via PPP and glycolysis, respectively. Subsequently, pyruvate formation through the tricarboxylic acid (TCA) cycle and mitochondrial oxidative phosphorylation were inhibited. These processes contributed to oxidative stress and ATP depletion, which upregulated the expression of Bax and Bak, led to the release of cytochrome c from mitochondria, activated caspase-3, and promoted PARP-induced membrane blebbing and cell apoptosis [8–10]. A high expression of SLC7A11 combined with glucose starvation or blockade of glucose uptake led to a high uptake of cystine, reduction to cysteine, NADPH depletion, and intracellular accumulation of disulfides, eventually causing disulfide stress. This stress activated the Rac–WRC-Arp2/3 signaling pathway, contributing to aberrant disulfide bonds in actin cytoskeleton proteins and disulfidptosis (Fig. 1).

Liu et al. [3] treated SLC7A11^high^ tumor cell lines with diverse cell death (apoptosis, autophagy, and ferroptosis) inhibitors and reducing agents (dithiothreitol (DTT), β-mercaptoethanol (2ME), and tris-(2-carboxyethyl)-phosphine (TCEP)) under glucose starvation conditions. Interestingly, the reducing agents rather than the cell death inhibitors completely reversed glucose starvation-induced SLC7A11^high^ cell death; however, this effect was not observed in SLC7A11^low^ or *SLC7A11* knockout (KO) tumor cells. Subsequently, a set of experiments, including the knockdown of ferroptosis and apoptosis-associated genes, was performed to further validate the mechanism of cell death manner unusual as pioneering studies. Thiol-oxidizing agents such as diamide and diethyl-maleate increased disulfide stress, resulting in SLC7A11^high^ cell death under glucose deprivation; however, inhibition of disulfide accumulation had the opposite effect [11]. *SLC7A11* deletion completely abolished diamine-induced SLC7A11^high^ cell death. These findings revealed that SLC7A11^high^ was a pivotal factor in mediating tumor cell death under glucose starvation conditions. Additionally, intracellular accumulation of disulfide molecules resulted in cell death via cystine and glutamyl-cystine [12], independent of ATP levels.

To analyze the expression of genes that were affected by disulfide accumulation in SLC7A11^high^ cells under glucose deprivation conditions, chemical proteomics and Gene Ontology (GO) enrichment analyses were performed. Glucose starvation triggered the formation of multiple intermolecular disulfide bonds in actin cytoskeleton proteins, which was an important factor mediating cell death, instead of NADPH deficiency. Furthermore, the clustered regularly interspaced short palindromic repeats (CRISPR)-CRISPR associated protein 9 (Cas9) technology was used to analyze whether knockdown of *SLC7A11* combined with cystine deprivation could suppress disulfidptosis in SLC7A11^high^ cells under glucose starvation condition; the results suggest that the formation of disulfide bonds in actin cytoskeleton proteins was indispensable for SLC7A11-mediated cystine import. Moreover, 2-deoxyglucose (2DG) promoted the generation of NADPH through the PPP and reversed disulfidptosis under glucose starvation in SLC7A11^high^ cells. Additionally, reactive oxygen species (ROS) scavengers could not inhibit disulfidptosis.

To clarify the association between disulfide bond formation and alteration of cell membrane dynamics, fluorescent staining of the actin filament (F-actin) and membrane was performed. Aberrant disulfide bond formation was noted in the actin cytoskeleton, which induced F-actin contraction and detachment from the plasma membrane, leading to morphological changes (cell shrinkage) and death in SLC7A11^high^ cells under glucose starvation. Furthermore, to study the regulation of disulfidptosis whole-genome CRISPR-Cas9 screening was performed, and several key genes were identified, including *SLC7A11*, *SLC3A2*, *RPN1*, and *NCKAP1* that encodes Nck-associated protein 1, an integral component of the WAVE regulatory complex (WRC), which activates actin-related protein 2 and 3 (Arp2/3) complex to promote actin polymerization and lamellipodia formation [13]. Knockdown of these genes attenuated disulfidptosis. Moreover, Rac activated the WRC to promote lamellipodia formation, which facilitated disulfidptosis.

Disulfidptosis was noted under glucose starvation conditions; therefore, the authors believed that blocking glucose transport (GLUT) would also have the same effect. To test this, they used GLUT inhibitors and observed disulfide bond formation in actin cytoskeleton proteins, F-actin contraction, and detachment from the plasma membrane in SLC7A11^high^ tumor cells, which ultimately resulted in cell death similar to the effects noted with glucose starvation. To extend the relevance of these findings to in vivo settings, SLC7A11^high^ tumor cell line and patient-derived tumor cells were used to establish two xenograft tumor models in vivo, followed by treatment with GLUT inhibitors, which significantly promoted disulfidptosis and inhibited tumor growth without side effects. However, this effect was limited to SLC7A11^high^ tumor cells with no effect on SLC7A11^low^ tumor cells.

Altogether, these novel findings are critical for expanding our knowledge of how disulfide stress induces cell death. The findings not only show the association between cellular metabolism and cell fate, but also highlight the mechanism regulating tumor cell disulfidptosis and may contribute to the development of a new therapeutic strategy against cancer. It is well established that groundbreaking findings lay a solid foundation for future investigation, and this work is no exception. Several aspects need to be studied further. First, it is crucial to determine whether pathways beyond the reported Rac–WRC-Arp2/3 signaling axis are involved in mediating disulfidptosis and the associated hallmarks. Second, the expression of SLC7A11 is not essential in disulfidptosis. Cell metabolism is a crucial factor, and whether disulfidptosis can be triggered under other conditions needs to be investigated. Third, whether disulfidptosis occurs in normal cells that possess self-repairing systems to maintain intracellular homeostasis, or whether disturbing this balance could contribute to diseases needs to be studied. Finally, from a therapeutic perspective, this study suggests that GLUT inhibitors have potential for use in cancer treatment; it is important to understand whether other target molecules exist.

## Conclusions

The discovery of disulfidptosis provides a new avenue for anticancer treatments that target the pathophysiological role of disulfide stress. The safety and efficacy of translational medicine targeting disulfidptosis need to be studied in several types of human cancers.

## Electronic supplementary material

Below is the link to the electronic supplementary material.

Supplementary Material 1

Supplementary Material 2

## Data Availability

The data supporting the findings of this study are available from the corresponding author upon reasonable request.

## Abbreviations

SLC7A11: solute carrier family 7 member 11
GO: Gene Ontology
GLU: glucose
HK: hexokinase
PPP: pentose phosphate pathway
G6P: glucose-6-phosphate
R5P: ribulose-5-phosphate
Cyto c: cytochrome c
TCA: tricarboxylic acid
Bax: Bcl-2-associated X protein
BAK: BCL2 antagonist
NADPH: nicotinamide adenine dinucleotide phosphate
Casp-3: caspase-3
PARP: poly (ADP-ribose) polymerase
WRC: WAVE regulatory complex
Apr: actin-related protein
