# Supplementary material for: Disulfidptosis: a new form of programmed cell death
Source: J Exp Clin Cancer Res. 2023 May 31;42:137. doi: 10.1186/s13046-023-02712-2 (PMC10230712; doi:10.1186/s13046-023-02712-2)
Supplement: Supplementary file 2 — Supplementary Material 2 [file 13046_2023_2712_MOESM2_ESM.docx]

**Response to commentary for** **Disulfidptosis: a new form of programmed cell death (JECC-D-23-00739R1)**

Dear Editor and reviewers:

Thank you for your thoughtful and useful review of our manuscript and the opportunity to further improve our work by revision. We have made all the required changes according to the suggestion by the reviewers. Please find our point-by-point responses to all the inquiries below.

**Reviewer #1:** In this commentary, the authors analyze disulfidptosis, a newly discovered programmed death, in detail. They introduce the mechanism of disulfidptosis and key experimental steps. However, I disagree with the author's proposed mechanism for disulfidptosis. The author lists three hallmarks necessary for its occurrence: (1) high expression of SLC7A11, (2) glucose starvation conditions, and (3) failure of the complex repair system that inhibits excessive intracellular disulfides and maintains homeostasis. In my view, depletion of NADPH, disulfide stress, and changes in the cytoskeleton are the key hallmarks of Disulfidptosis. Boyi Gan et al.'s original text also introduced an induction pathway for Disulfidptosis unrelated to (1) and (2). For instance, direct administration of high concentrations of cystine in the culture environment can induce Disulfidptosis in cells with low SLC7A11 expression. Two articles related to disulfidptosis (PMID: 36918690, 37101248) are recommended for reference.

Response: Thank you very much for your constructive opinions and useful review of our manuscript to further improve our work by revision. We are carefully read the Boyi Gan et al.'s original text again, and reference of PMID: 36918690, 37101248, we are strongly agree with your opinion that depletion of NADPH, disulfide stress, and disulfide bonds between actin cytoskeleton proteins are the key hallmarks of disulfidptosis. The high expression of SLC7A11 is not indispensable during the cell disulfidptosis. High SLC7A11 expression will increase cysteine contribution to disulfide stress; glucose starvation will limit PPP and depletion of NADPH, these alteration result in disulfide bonds between actin cytoskeleton proteins, eventually leading to disulfidptosis. We have amended this issue. Please check the details below:

On page 2, lines 56,

The activation of disulfidptosis might require three hallmarks:

On page 2, lines 59,

accumulation of cysteine contribute to disulfide stress in cell metabolism

On page 2, lines 62-63

3) formation of aberrant disulfide bonds between actin cytoskeleton proteins.

**Reviewer #2:** The authors have addressed most of the raised concerns.

Response: Thanks very much for your positive evaluation of our work.

Finally, we appreciate for Editor/Reviewers’ warm work earnestly and hope that the correction will meet with approval. Once again, thank you very much for your comments and suggestions.

Best regards,

Yours sincerely,

Chong Zeng

Medical Research Center, Shunde Hospital, Southern Medical University

Foshan, Guangdong, China, 528300

T: +86 757 22318169

E-mail: wonder10@126.com
